# Deep Convolutional Neural Network for EEG-Based Motor Decoding

**DOI:** 10.3390/mi13091485

**Published:** 2022-09-07

**Authors:** Jing Zhang, Dong Liu, Weihai Chen, Zhongcai Pei, Jianhua Wang

**Affiliations:** 1 School of Automation Science and Electrical Engineering, Beihang University, Beijing 100191, China; 2 Center of Artificial Intelligence, Hangzhou Innovation Institute, Beihang University, Hangzhou 310051, China; 3 ByteDance, Hangzhou 311100, China

**Keywords:** brain–machine interface (BMI), electroencephalography (EEG), convolutional neural network (CNN), motor decoding

## Abstract

Brain–machine interfaces (BMIs) have been applied as a pattern recognition system for neuromodulation and neurorehabilitation. Decoding brain signals (e.g., EEG) with high accuracy is a prerequisite to building a reliable and practical BMI. This study presents a deep convolutional neural network (CNN) for EEG-based motor decoding. Both upper-limb and lower-limb motor imagery were detected from this end-to-end learning with four datasets. An average classification accuracy of 93.36 ± 1.68% was yielded on the four datasets. We compared the proposed approach with two other models, i.e., multilayer perceptron and the state-of-the-art framework with common spatial patterns and support vector machine. We observed that the performance of the CNN-based framework was significantly better than the other two models. Feature visualization was further conducted to evaluate the discriminative channels employed for the decoding. We showed the feasibility of the proposed architecture to decode motor imagery from raw EEG data without manually designed features. With the advances in the fields of computer vision and speech recognition, deep learning can not only boost the EEG decoding performance but also help us gain more insight from the data, which may further broaden the knowledge of neuroscience for brain mapping.

## 1. Introduction

The past decade has witnessed a vigorous development of brain–machine interfaces (BMIs) for communication and rehabilitation [1]. A BMI is a pattern recognition system that translates brain activities into messages and control commands bypassing peripheral somatomotor nervous system. The brain signal is usually acquired or measured by a variety of invasive or non-invasive techniques, most notably, electroencephalography (EEG). With the great advantage of non-invasive, high time resolution, fairly low-cost, and easy adaptability to external devices, EEG has been widely investigated in both the academic community and in clinical trials. Typical implementations include brain-actuated mobile robots [2], brain-controlled wheelchairs [3], robot-assisted gait training [4], and EEG-based robotic arms [5].

EEG-based BMIs can be categorized into the evoked paradigm, i.e., exogenous brain signals were used in the decoding framework, and spontaneous paradigm, i.e., endogenous brain signals were employed for the detection. For the former, the subject needs to be presented with external stimuli, e.g., visual or auditory cues, which might limit the flexibility of the application and lead to fatigue after a long attendance. The steady state visually evoked potential (SSVEP) and P300 are the most commonly used EEG correlates in these BMIs. On the other hand, spontaneous signals, e.g., sensorimotor rhythms (SMRs), also known as motor imagery (MI), and slow cortical potentials (SCPs) are exploited in the spontaneous BMI with a natural and asynchronous framework where the subject is able to voluntarily operate the external devices. Moreover, these asynchronous BMIs are more likely to promote the active participation of the human nervous system which has been shown to induce brain neural plasticity and thus to enhance the likelihood of motor recovery. The drawback is the relatively long training procedure and low information transmission rate. Nevertheless, MI-based BMI is a very promising technology with an intuitive decoding architecture to directly control external devices.

Designing a practical BMI is a complex task which requires multidisciplinary knowledge in neuroscience, signal processing, computer science, and engineering [6,7]. In order to build an asynchronous BMI, offline training and online testing are two basic steps, which are generally composed of signal acquiring, preprocessing, feature extraction, classification, decision making, and feedback. While the ultimate goal is to obtain a reliable and robust perception-action closed loop, machine learning techniques play a central role in the decoding pipeline to address the issue of poor signal-to-noise ratio. In recent years, machine learning algorithms have been widely adopted in BMI systems to learn and decode different brain patterns. For instance, features were extracted from power spectral density and feature selection was conducted based on canonical variate analysis in [8]. A Gaussian mixture model was used to classify motor imagery of the left hand, the right hand, and both feet as a control interface for motor disabled users. Another work used a similar framework to control a self-balanced powered exoskeleton [9], while the classifier was a random forest model which was a bagging model and had good generalization capacity. A review of brain-controlled mobile robots and their implemented classification methods can be found in [10]. The SMR-based BMI has been one of the fastest growing areas with clinical applications [11,12].

More recently, deep learning, as a branch of machine learning, has overcome previous limitations in various areas, e.g., image classification, speech recognition, and natural language processing [13,14,15]. Compared with images and text, EEG data is much more difficult to obtain. However, thanks to the dedicated effort by several research groups [16,17], more and more public datasets can be accessed to alleviate this issue. In decoding the EEG signals, traditional machine learning techniques, such as support vector machine and linear discriminant analysis, relied heavily on knowledge provided by neuroscience, which implies that data preprocessing and manual feature selection were necessary for these frameworks [18]. For instance, MI was decoded in the frequency domain (8–12 Hz and 14–30 Hz), and the features were distributed around C3, C4, and Cz. It is realized that manual feature designing hardly works ina certain scenario where little prior knowledge is available for the EEG correlates. These scenarios include hybrid EEG features, e.g., combination of SCP and MI [19], and new mental tasks, e.g., lower-limb MI of extension and flexion [20,21]. To transfer from hand-designed feature extraction to data-driven approach, representation learning, particularly deep learning which has shown great promise [22], is considered in this work in EEG decoding.

A deep learning framework can discover discriminative features for the classification of mental tasks without manual feature design. Complex mapping from raw data to the labels can also be realized by deep learning, which is based on multiple nonlinear layers. Compared with deep neural network with multilayer perceptron, convolutional neural network (CNN) has the advantage of local connection and shared weights, and therefore largely reduces the complexity of network structure. In recent years, convolutional neural networks have gained extensive attention because of their excellent performance in electroencephalogram (EEG) [23,24]. For motor imagery EEG data, Yang et al. [25] developed an end-to-end CNN framework with central distance loss to improve the discrimination of features and classification performance. Furthermore, Li et al. proposed a multi-scale fusion convolutional neural network based on an attention mechanism (MS-AMF) and verified the effectiveness in the BCI competition IV-2a dataset [26]. Moreover, a novel end-to-end MI-EEG decoding framework temporal-spectral-based squeeze-and-excitation feature fusion network (TS-SEFFNet) was proposed on two public MI-EEG datasets and received promising results in accuracy [27].

In this work, we performed EEG-based motor decoding with a deep learning framework. A large amount of an EEG dataset was first collected from public data sources and refined. We further designed experimental protocols and executed recordings to acquire EEG data. Moreover, we proposed a CNN-based data processing method to classify mental tasks, e.g., MI of the left hand vs. the right hand and MI of lower-limb extension vs. flexion. This CNN-based method was compared with two state-of-the-art algorithms, namely, common spatial patterns (CSP) + support vector machine (SVM), and deep neural network (refers to multilayer perceptron in this work). Finally, we show the discriminative features and brain pattern modulations learned by the proposed method. To the best of our knowledge, this is the first study using a CNN-based framework to perform both upper-limb and lower-limb motor decoding. The proposed approach provides an alternative for feature selection and classification for EEG analysis, as well as a new way to build a practical BMI. The main contribution of this work is to present a deep CNN-based framework to decode EEG-based motor imagery for both upper-limb and lower-limb applications. Our results suggest that the CNN-based framework has an advantage over two other typical methods, which could be used to improve the performance of a BMI for motor training and functional recovery.

## 2. The Datasets

In this work, the data come from four sources, including a public dataset and customized recordings in our lab. The first dataset is the PhysioNet’s online database [16], which was created and collected by the developers of the BCI2000 [28]. It consists of 1526 one- and two-minute EEG recordings obtained from 109 subjects. Each participant performed 14 runs of recordings using a 64-channel (the international 10/10 system) EEG measurement system with the sampling frequency of 160 Hz. The mental task was MI of opening and closing left or right fist, which was commonly used in asynchronous BMIs.

The second dataset is taken from the BCI competition III dataset IVa. Five subjects (280 trials per subject) participated in the recording to perform MI of the left hand, the right hand, and the right foot. EEG data were recorded by BrainAmp amplifiers with 118 EEG channels at 1000 Hz.

**Figure 1:**
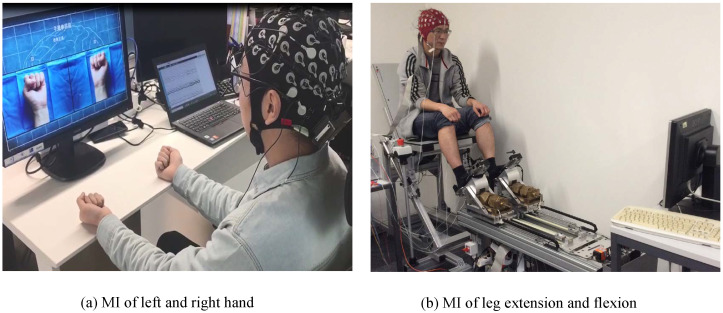
Snapshot of the two experimental scenarios with MI of the left hand vs. the right hand and MI of leg extension vs. flexion, which generate the third and the fourth dataset, respectively.

The third dataset was collected in our lab (Intelligent Robot and Measurement and Control Technology laboratory, IR and MCT). Eight volunteers (four female, age 25.7 ± 1.5) participated in the experiment. The mental task was 4 s MI of the left hand and the right hand, which was cued by a visual presentation using a customized Python openCV script (see Figure 1a). The experimental session consisted of 5 runs, and each run was composed of 60 trials. EEG signals were acquired by a portable Neuracle system (www.neuracle.cn (accessed on 31 August 2022)) with 64 electrodes arranged in the modified 10/20 international system sampling at 1000 Hz.

Finally, the fourth dataset was produced from lower-limb motor imagery experiments carried out in a Defitech Chair in Brain–Machine Interface (CNBI) [20]. Nine volunteers (five female, age 23.33 ± 1.87) participated in the experiment, and each of them conducted three recording sessions (see Figure 1b). Each session was composed of 5 runs and each run consisted of 60 trials with extension and flexion cues balanced and randomized inside. EEG signals were acquired by a portable BioSemi ActiveTwo system (www.biosemi.com (accessed on 13 July 2022)) with 32 electrodes arranged in the modified 10/20 international system sampling at 2048 Hz. A comparison of the four dataset can be found in Table 1.

## 3. Data Processing

As the EEG data was collected by different devices, we cannot pool them together. In this work, we processed each of the four datasets separately, taking the fourth dataset as our example to show the processing pipeline. We then compared the CNN-based framework with two other state-of-the-art methods, i.e., SVM-based method [29] and DNN-based framework [30,31].

### 3.1. CNN-Based Framework

The CNN is a specialized kind of neural network that can process data which has a grid-e topology. Similar to images, we treated EEG data as 2D grids of pixels, while the height is equal to one (such as a grey image). The overall MI period in one trial was 4 s. The EEG data were first downsampled from 2048 to 512 Hz. We took the period of [0, 2] s after the visual cue and over all 32 channels as the training data. Therefore, the size of each training sample is like an image with the length of 32 and the width of 1024. The total training samples were 9 (subjects) × 3 (sessions) × 300 (trials). Instead of hand designed features (feature extraction and feature selection), we used the raw data to feed the classifier. Consequently, we can validate whether a generic CNN could reach a competitive accuracy without any prior neuroscience knowledge.

**Figure 2:**
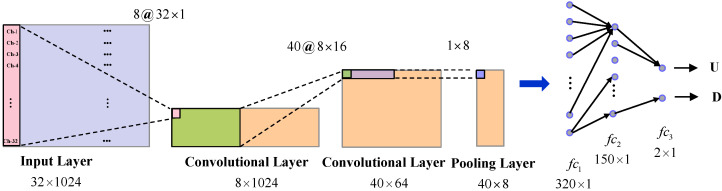
The architecture of the proposed CNN−based framework for the classification of lower−limb MI. U and D refer to lower−limb extension (**up**) and flexion (**down**), respectively.

The architecture of our network is shown in Figure 2. It contains three hidden layers: two convolutional layers with max pooling and another fully connected layer. The output of the last fully connected layer was fed to two neurons which produced the two class labels (up and down).

The first convolutional layer filtered the 32 × 1024 input EEG signal with eight kernels of size 32 × 1 with a stride of one sample. The second convolutional layer took as input the output of the first convolutional layer 8 × 1024 and filtered it with 40 kernels of size 8 × 16. Then a max pooling layer was added to the second layer with the kernel of 1 × 8. The third fully-connected layer had 150 neurons, and the last layer was also a fully-connected neurons as output.

Other parameters and settings include: (1) The activation function of the first three layers was the Rectified Linear Units (ReLUs), which had been proved to train several times faster than sigmoid or tanh units. Relu was also beneficial to overcome the vanishing gradient problem. The last layer with fully connection used the softmax activation function. (2) The loss function was cross entropy rather than square error, which was easier to train. (3) Max pooling was used in the pooling layer, which summarized the outputs of neighboring groups of neurons in the same kernel map. (4) The training process was based on backpropagation, but with an adaptive learning rate. In this work, we used Adam (a combination of advanced adagrad and RMSprop) as the optimizer to perform the training as follows,

(1) $$\begin{array}{c} V_{dW}=\beta_{1}V_{dW}+\left(1-\beta_{1}\right)dW\\ S_{dW}=\beta_{2}S_{dW}+\left(1-\beta_{2}\right)dW^{2}\\ V_{dW}^{corrected}=\frac{V_{dW}}{1-\beta_{1}^{t}}\\ S_{dW}^{corrected}=\frac{S_{dW}}{1-\beta_{2}^{t}}\\ W:=W-\alpha \frac{V_{dW}^{corrected}}{\sqrt{S_{dW}^{corrected}+\varepsilon }} \end{array}$$

where $\beta_{1}$ was set to 0.9, $\beta_{2}$ was set to 0.999, and ε was set to 1 × 10${}^{-8}$. There were hyperparameters that needed to be manually set. The most essential hyperparameter was the learning rate α which needed to be carefully chosen. Furthermore, $V_{dW}$ and $S_{dW}$ were initialized to 0, and *t* was the iterate step. All the optimization was performed on the mini batch.

We used a desktop configured with core i5 (2.5 GHz), 16 GB RAM and NVIDIA GTX 1070 3 GB GPUs for the data processing. The models were trained using stochastic gradient descent with a batch size of 128 examples. The parameters of the network were the weights and biases. The weights were initialized in each layer using a zero-mean Gaussian distribution with standard deviation 0.01. The biases were initialized in each layer with the constant one. This kind of initialization can accelerate the early stages of learning by providing the ReLUs with positive inputs. Furthermore, we used an equal learning rate for all layers. Learning rate was the most important hyperparameter to tune. We adjusted the learning rate manually throughout training. We followed a heuristic rule that if the validation error rate stopped improving with the current learning rate, we divide the learning rate by three. The learning rate was initialized at 0.0001 and reduced three times prior to termination.

### 3.2. Comparison with State-of-the-Art Methods

**Figure 3:**
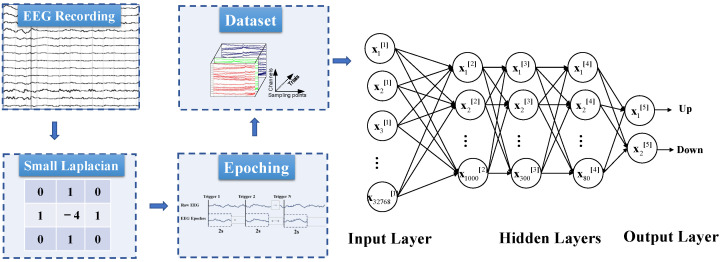
DNN-based framework for the decoding. U and D refer to lower-limb extension (**up**) and flexion (**down**), respectively.

In addition to the CNN-based framework, we built two other models for the classification, i.e., DNN-based method and SVM-based approach. The DNN-based framework consisted of small Laplacian spatial filtering, epoching, and multilayer perceptron (MLP) with the architecture shown in Figure 3. The EEG data were first spatially filtered by a small Laplacian, where at each time point, the average amplitude over the nearest four orthogonal electrodes was subtracted from each channel. The aim of this process was to remove the global background activity and enhance the signal-to-noise ratio (SNR). Then the signals were down-sampled to 512 Hz and segmented into 2 s epochs, resulting in 32 × 1024 × 1 image-like samples. The neural network contains three hidden layers and the number of neurons in each layer is 1000, 300, 80. The output layer contains two neurons.

With a large number of parameters (weights and biases) to train, this model has a high risk of overfitting. The most useful strategy is to increase the training data. However, EEG data are much harder to obtain compared with images, texts, or speeches. To prevent overfitting, we used the optimization method commonly used in deep learning. For instance, we used L2 regularization also called weight decay. The loss function is

(2) $$C=-\frac{1}{n}\sum_{x_{j}}\left[y_{j}lna_{j}^{L}+\left(1-y_{j}\right)ln\left(1-a_{j}^{L}\right)\right]+\frac{\lambda }{2n}\sum_{w}w^{2}$$

where *n* is the number of training data, and *L* is the number of layers. The first term is the cross entropy, and the second term is the regularizaton term. Moreover, $y_{j}$ is the label of *j*th sample, and $a_{j}$ is the output of $y_{j}$ neuron. The regularizaton was only performed on the weights, and this term was modulated by the regularization parameter λ. The overall training was also based on backpropagation with a mini-batch method. We used RMSprop to train faster as follows,

(3) $$\begin{array}{c} S_{dW}=\beta S_{dW}+\left(1-\beta \right)dW^{2}\\ S_{db}=\beta S_{db}+\left(1-\beta \right)db^{2}\\ W:=W-\alpha \frac{\mathrm{dW}}{\sqrt{S_{dW}}}\\ b:=b-\alpha \frac{\mathrm{db}}{\sqrt{S_{db}}} \end{array}$$

where α was the learning rate which needs to be tuned, and β was set to 0.9. The parameters *W* and *b* are optimized based on gradient descent, and dW and db are partial derivatives with the loss function. The DNN-based framework is a hierarchical representation learning method. We use random choice to tune the hyperparameters and gradient checking to debug the model.

**Figure 4:**
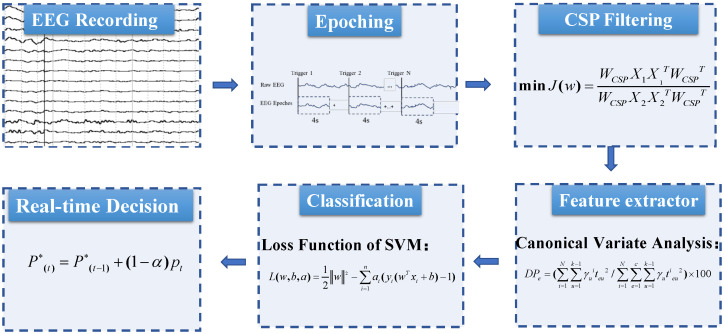
The architecture of the SVM-based framework for the decoding.

The other model we used in the current work was the SVM-based method (see Figure 4). Rather than representation learning, human-designed features were used as the input in the classifier. The EEG data were first processed by CSP, which was a supervised algorithm to maximise the variance of the signals for one class and minimise it for the other classes. CSP used the spatial filter $W_{CSP}$ to minimize the following function,

(4) $$J\left(w\right)=\frac{W_{CSP}X_{1}X_{1}^{T}W_{CSP}^{T}}{W_{CSP}X_{2}X_{2}^{T}W_{CSP}^{T}}$$

where $X_{i}$ was the training data matrix for class *i* with the shape of channel × samples. The function was a generalized Rayleigh quotient, and therefore extremising this function can be solved by Generalized Eigen Value Decomposition (GEVD). The data were then segmented into training and validation sets. We calculated the multitaper power spectral density with the window length of 1 s and shifted every 62.5 ms in the 4 s MI period. Then canonical variant analysis was conducted to extract canonical discriminant spatial patterns (CDSPs) whose directions maximize the separability in the features between the given classes. We manually selected the number of features for feature selection to prevent overfitting. Finally, we used SVM as the classifier with the loss function as,

(5) $$L\left(w,b,\alpha \right)=\frac{1}{2}{∥w∥}^{2}-\sum_{i=1}^{n}\alpha_{i}\left(y_{i}\left(w^{T}x_{i}+b\right)-1\right)$$

where $\alpha_{i}$ was the Lagrange multiplier for the ith training sample, *w* and *b* were the parameters of the model, and $x_{i}$ and $y_{i}$ were the training dataset and corresponding labels. Sequential minimal optimization (SMO) was applied to solve the optimization problem. Since a single window is not reliable due to the highly noisy nature of the EEG signals, we performed a smoothing procedure, also known as evidence accumulation, in order to obtain a more robust decision at the trial level [32]. The likelihood for each sample was integrated as:

(6) $$P_{\left(t\right)}^{*}=\alpha_{s}P_{\left(t-1\right)}^{*}+\left(1-\alpha_{s}\right)p_{t}$$

where $\alpha_{s}$ was the smooth factor, and it was set to 0.95 or 0.96 in our experiments based on the previous experience [8,20]. The BMI would respond as soon as the integrated likelihood surpassed a certainty threshold. The certainty threshold was usually chosen [0.70, 0.85] based on the performance.

### 3.3. Training Process and Feature Visualization

The CNN-based and DNN-based methods have similar training processes. The main goal is to decrease the training loss (cross entropy) through backpropagation with Adam and RMSprop, respectively. In addition to regularization, we also performed early stop to prevent overfitting. The hyperparameters, e.g., learning rate and neural network architecture, were tuned accordingly on each dataset. The parameters (weights and biases or values in the convolutional kernel) were saved for testing. Furthermore, feature visualization was conducted by averaging the weights along the timepoints, and the feature importance was shown by topographies.

For the SVM-based approach, feature analysis was conducted to evaluate any eventual difference in discriminant features between the two classes. We used the same CVA method to rank the features (channel and frequency pairs) employed for the classification. The most important hyperparameters for this method were the number of features. We performed a grid search to evaluate the validation error along different feature numbers. Feature visualization was further conducted with scalp distributions by averaging the discriminant power across the frequency bands. We compared the discriminative features employed by the CNN-based and the SVM-based method.

## 4. Results and Discussion

### 4.1. Decoding Performance

**Figure 5:**
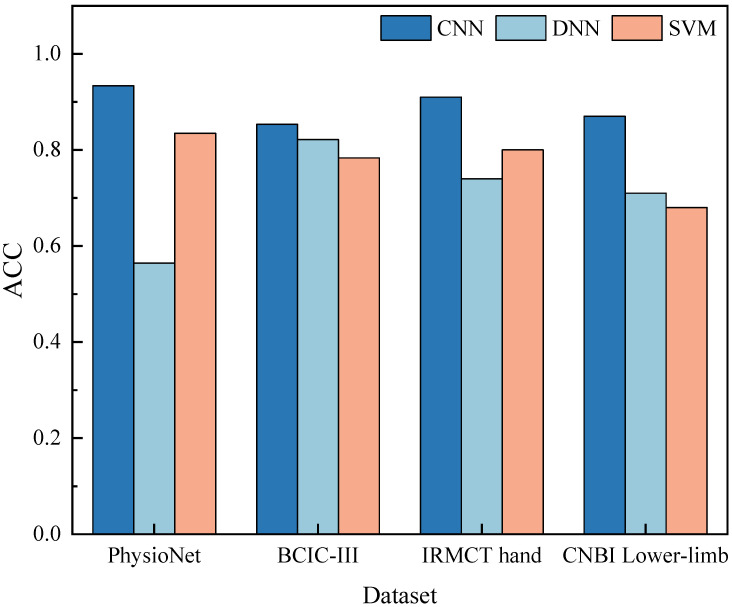
The classification accuracy (ACC) of the three decoding frameworks over the four datasets.

Figure 5 displays the classification performance of the three decoding frameworks over the four datasets. The classification accuracy of the CNN-based framework over 10-fold cross validation was 93.36 ± 1.68%. The ACC of the DNN-based and the SVM-based methods were 56.44 ± 3.82% and 83.46 ± 2.41%, respectively. We calculated the chance level based on binomial distribution [33], which yielded a chance accuracy of 56.67%. The classification accuracy of all three methods was significantly higher than the chance level (one-tailed two-sample t-test with all the *p* values smaller than 0.05, Bonferroni correction). The repeated measures ANOVA on the ACC with the factor decoding method (*p* < 0.05) reported a significant learning effect. Multiple comparisons with the Tukey–Kramer critical value showed that the performance using the CNN was significantly better than the performance of the other two methods.

For the second dataset, the mean ACC of the CNN, DNN and SVM-based methods were 85.32 ± 3.00%, 82.15 ± 7.20%, and 78.62 ± 8.17%. No statistically significant differences were found by the repeated measures ANOVA (*p* > 0.05) on the decoding performance. For both the IRMCT hand and lower limb dataset, significantly better performance was observed from the CNN-based method than the other methods. There were no significant differences between the DNN-based and the SVM-based method. The mean ACC of the CNN-based framework on these two datasets was 0.91 ± 3.80 and 0.87 ± 1.22, respectively.

### 4.2. Training Process and Feature Visualization

**Figure 6:**
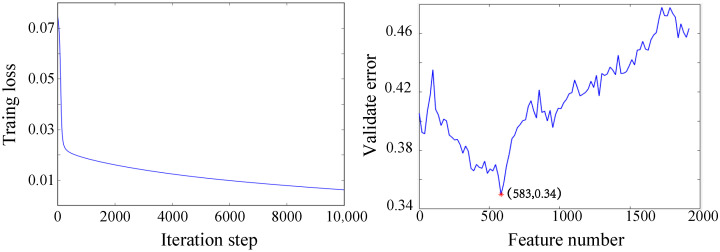
The training process of the CNN-based framework (**left panel**) and feature selection process of the SVM-based framework (**right panel**).

The training process of the CNN-based method is shown in Figure 6 (left panel). The overall loss reduced sharply at the starting iteration steps, indicating the feasibility of the proposed framework. The training loss was also the criteria for hyperparameter tuning. For instance, a relatively small learning rate might lead to a slight change of the loss, while a large learning rate might lead to the divergence. For a shallow network, such as the SVM, the number of features should be manually set. As shown in Figure 6 (right panel), the optimized number of features was 600.

**Figure 7:**
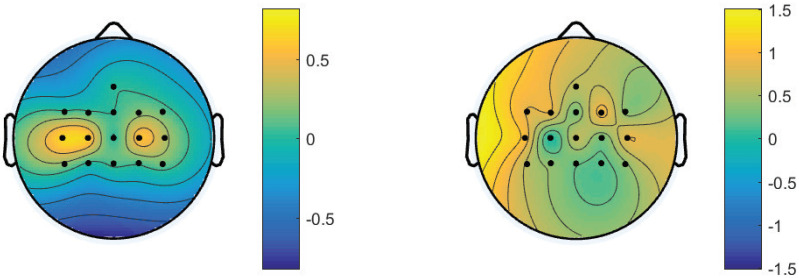
Scalp topographies to show the feature representation of the SVMbased method (**left panel**) and the CNN-based method (**right panel**) for the decoding of the left and right hand MI with 16 EEG channels.

For the classification of the left and right hand MI, the discriminative features exploited in the SVM and the CNN-based methods are shown in Figure 7 left and right panel, respectively. There was a visible difference between the topographic maps, illustrating that different features were used in the two models. For the SVM-based method, C3, C4, C1, and C2 were the most frequently selected channels. This is consistent with previous works in SMR-based BMI [2,8]. On the other hand, features selected by the CNN-based method were distributed along all channels. The neural network extracted information from various channels without prior knowledge. It is worth noting that there was no time-frequency transformation for the CNN-based method, although highest accuracy was obtained by this model.

**Figure 8:**
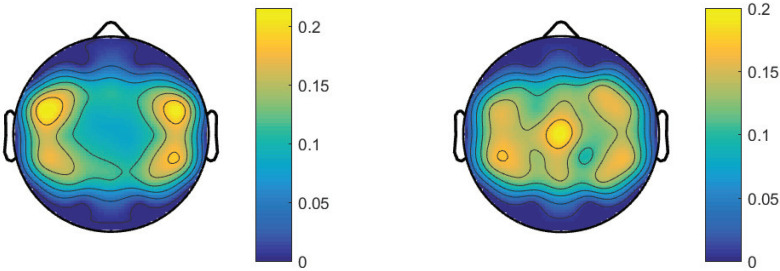
Scalp topographies to show the feature representation of the SVM-based method (**left panel**) and the CNN-based method (**right panel**) for the decoding of lower-limb extension and flexion MI with 32 EEG channels.

Figure 8 presents the discriminative features used by the two models for MI of lower-limb extension and flexion. More focal features were distributed at FC5, FC6, CP5, and CP6 for the SVM-based model. This is consistent with the previous works using the random forests classifier [20,34]. For the CNN-based method, features were mainly located at the middle areas. The most significant difference between the two models was the features distributed at Cz. Similar to the CNN-based hand MI decoding, no feature extraction and feature selection were performed for the classification. This indicates that a deep learning approach might help to gain more insight from data.

In this work, we developed a CNN model to perform the classification on both upper-limb and lower-limb MI with four datasets. The proposed neural network can map a sequence of EEG samples to different class labels. An average classification accuracy of 93.36 ± 1.68% was yielded on a large dataset with 109 subjects. We further compared this model with two other models, i.e., multilayer perceptron and the state-of-the-art SVM-based approach. The performance using the CNN was significantly better than the performance of the other two methods. Finally, we showed the training process and feature selection of the two frameworks. Feature visualization was conducted to show the discriminative channels for the classification.

Compared with multilayer perceptron which is a standard feedforward neural network with similarly-sized layers, the CNN has much fewer connections and parameters; therefore, it is easier to train. In the meantime, the theoretically best performance of the CNN is likely to be only slightly worse than multilayer perceptron. In contrast to images, texts, and speeches, EEG data are much harder to obtain, making the training dataset insufficient to train a big model. Although various strategies, e.g., regularization and early stop, were used in this work to prevent overfitting, the relatively low sample-to-feature ratio is still a non-negligible challenge for the deep learning approach. For the decoding accuracy, the CNN performed much better than multilayer perceptron with the same number of layers. While the CNN is an active research field presently, attention should be drawn to bring more advanced or sophisticated neural network architecture in computer vision or natural language processing to EEG-based motor decoding. For instance, the previously published ResNet architecture largely reduced the error rates on the ImageNet image-recognition challenge, where 1.2 million images must be classified into 1000 different classes, from above 26% to below 4% within 4 years [35]. More recently, the Dense Convolutional Network was proposed which has the advantage of alleviating the vanishing-gradient problem and strengthening feature propagation [36]. A traditional processing framework usually has a step for feature extraction to glean useful information from the signals [8,20,37]. Raw EEG signals were used to train the neural network in the current work. No prior neuroscience or neuroengineering knowledge was necessary for the decoding. The nonlinearity of the network model can approximate arbitrary function, thus mapping the raw EEG input into training labels. Compared with the traditional shallow model, the disadvantage of the deep learning approach is the lack of interpretation in the feature domain. Especially for motor imagery, discriminant power can be shown in both frequency bands and channels. Furthermore, the training sample in the current work was a 2 s time window in the MI period, while for the SVM-based method, sliding windows were used to extract the features in 500 ms.

The current protocol can be extended to perform multiple types of mental tasks, e.g., MI of the left hand, the right hand, both feet and the rest as fourth classes. For instance, subjects can modulate their sensorimotor rhythms to control an AR drone navigating a 3D physical space [38]. In this paradigm, imagining use of the right hand turned the quadcopter right and imagination of the left hand turned it left. Imagining both hands together caused the helicopter to rise, while intentionally imagining nothing caused it to fall. we have conducted the testing with four classes. However, the performance was close to chance level. The subject needs more training to learn to adjust their mental strategies. Moreover, other spontaneous EEG signals, namely, movement-related cortical potentials (MRCPs) have been decoded as a brain switch to trigger external robotic devices [39,40,41]. The current framework can be used in these scenarios for neuromodulation. In previous works concerning EEG decoding using deep learning approach, a Bayesian framework was proposed in [42] where the class-discriminative frequency bands are probabilistically selected and the corresponding spatial filters are optimized. A backpropagation-based joint optimization methodology was proposed in [43] as a wrapper to fine-tune the parameters with a limited number of samples. They showed that the deep neural network could still have interpretable components. Moreover, a deep CNN was proposed in a recent work with a range of different architectures, designed for decoding imagined or executed movements from raw EEG [44]. They proved that recent advances from the machine learning field, including batch normalization and exponential linear units, together with a cropped training strategy, boosted the deep CNN decoding performance, reaching or surpassing that of the widely-used filter bank common spatial patterns (FBCSP) decoding algorithm.

The main contribution of the CNN-based framework is the temporal representation. Recent work can be found in [45], with the representation generated from modifying the filter-bank common spatial patterns method. Temporal characteristics of EEG were gained by studying the convolutional weights of the neural networks. In addition to motor imagery, this framework can be also used in exogenous brain patterns, e.g., steady-state visual evoked potential [46]. A P300 speller was built in [47] based on the CNN with equivalent accuracy to the best method on the third BCI competition Data Set II but outperformed the best method when the EEG channels reduced from 64 to 8 and the considered epoch is 10. The classifier does not consider high level features as input and provides tools for interpreting brain activities. A recent work conducted by Ng’s group also used the CNN to perform arrhythmia detection from electrocardiograms (ECG) [48]. They trained a 34-layer CNN mapping a sequence of ECG samples to a sequence of rhythm classes and realized a cardiologist-level diagnosis.

Another important issue is the feedback in the BMI framework. Feedback is necessary for the subject to actively control the external device in a closed loop. The feedback, which is linked directly to the perceptual processes and the brain patterns, would enable the subject to learn to modulate his/her brain activities to obtain a more effective training. Moreover, performing the mental task with feedback can send back the modulation of the sensorimotor rhythm (SMR) through a different pathway, which might lead to brain plasticity, rewiring, and rehabilitation. Further work would be executed on an online control task to provide feedback to the user in real time.

## 5. Conclusions

This paper has presented EEG-based motor decoding with a deep CNN-based framework for both upper-limb and lower-limb implementations. Significantly better performance was observed in the current study compared with two other typical methods. We also conducted feature visualization to evaluate the discriminate power in the decoding. The preliminary results in this work have shown the feasibility and superiority of deep learning in BCI applications. Further work would be conducted with extended datasets, various neural network architectures, and transfer learning trained across multiple subjects and refined on individuals. In addition to EEG, we would try the deep CNN method to learn features from intracortical or ECoG recordings. The signal-to-noise ratio of these invasive signals is significantly higher than EEG. Consequently, fundamental truths about the brain’s processing might be revealed from the decoding framework. Finally, all the datasets and recordings are from healthy subjects, while the ultimate users of the proposed system will be patients with motor disorders. Future work will be devoted to collect the EEG (as well as ECoG) data from patients.

## Figures and Tables

**Table 1:** Data sources used in this work.

Source	Subject Number	Mental Tasks	EEG Channels	Sampling Frequency
PhysioNet Movement				
Imagery Dataset	109	MI of left/right hand	64	160 Hz
BCI competition III				
Dataset IV-a	5	MI of right hand/foot	118	1000 Hz
Experiment in				
IRMCT lab	8	MI of left/right hand	16	512 Hz
Experiment in				
CNBI lower-limb	9	MI of leg extension/flexion	32	2048 Hz

## Data Availability

The authors sincerely thank motion imagery data of PhysioNet’s online database at https://physionet.org/about/database/ (accessed on 13 July 2022) BCI competition III dataset IVa at http://www.bbci.de/competition/iii/ (accessed on 13 July 2022) provided by the Institute for Knowledge Discovery (Laboratory of Brain-Computer Interfaces), Graz University of Technology, Graz, Austria (Clemens Brunner, Robert Leeb, Gernot Müller-Putz, Alois Schlögl, Gert Pfurtscheller) for their data providing.

## Abbreviations

The following abbreviations are used in this manuscript:

BMI	brain–machine interface
CNN	convolutional neural network
EEG	electroencephalography
SVM	support vector machine
CSP	Common Spatial Pattern
CVA	Canonical Variate Analysis
